# The Functions of an NAC Transcription Factor, GhNAC2-A06, in Cotton Response to Drought Stress

**DOI:** 10.3390/plants12213755

**Published:** 2023-11-02

**Authors:** Gulisitan Saimi, Ziyu Wang, Yunhao Liusui, Yanjun Guo, Gengqing Huang, Huixin Zhao, Jingbo Zhang

**Affiliations:** Xinjiang Key Laboratory of Special Species Conservation and Regulatory Biology, College of Life Science, Xinjiang Normal University, Urumqi 830054, China; 13345443145@163.com (G.S.); wzy18699315627@163.com (Z.W.); liusuiyunhao@163.com (Y.L.); coad25@foxmail.com (Y.G.); gqhuang@mail.ccnu.edu.cn (G.H.)

**Keywords:** NAC, cotton, drought stress, function, expression

## Abstract

Drought stress imposes severe constraints on crop growth and yield. The NAC transcription factors (TF) play a pivotal role in regulating plant stress responses. However, the biological functions and regulatory mechanisms of many cotton NACs have not been explored. In this study, we report the cloning and characterization of *GhNAC2-A06*, a gene encoding a typical cotton NAC TF. The expression of *GhNAC2-A06* was induced by PEG treatment, drought stress, and ABA treatment. Furthermore, we investigated its function using the virus-induced gene silencing (VIGS) method. *GhNAC2-A06* silenced plants exhibited a poorer growth status under drought stress conditions compared to the controls. The *GhNAC2-A06* silenced cotton plants had a lower leaf relative water and chlorophyll content and a higher MDA content compared to the controls under the drought treatment. The levels of superoxide dismutase (SOD), peroxidase (POD), and catalase (CAT) enzyme activity in the *GhNAC2-A06* silenced plants were found to be lower compared to the controls when exposed to drought stress. Additionally, the downregulation of the drought stress-related genes, *GhSAP12-D07*, *GhNCED1-A01*, *GhLEA14-A11*, *GhZAT10-D02*, *GhPROT2-A05*, *GhABF3-A03*, *GhABF2-D05*, *GhSAP3-D07*, and *GhCPK1-D04*, was observed in the *GhNAC2-A06* silenced cotton. Together, our research reveals that *GhNAC2-A06* plays a role in the reaction of cotton to drought stress by affecting the expression of genes related to drought stress. The data obtained from this study lay the theoretical foundation for further in-depth research on the biological function and regulatory mechanisms of *GhNAC2-A06*.

## 1. Introduction

With the increase in global temperature, the risk of drought-related disasters is also increasing [1]. Drought has a far-reaching impact on the growth and development of crops, seriously affecting their yield and quality [2]. To mitigate and adapt to drought stress, plants have developed a comprehensive array of signal transduction systems. These include sensors, secondary messengers, hormones, and signal receptors. These signal components transmit drought signals to the cells and regulate the expression levels of stress-related genes, ultimately causing plants to undergo physiological and morphological changes to counteract drought stress [3]. Plant transcription factors play vital roles in plant resistance to drought, directly regulating the expression of drought-inducible genes [4]. To date, many drought-stress-regulating plant transcription factors, including AP2, bZIP, NAC, WRKY, and MYB, have been identified [5].

The NAC transcription factor is the largest known family of plant-specific transcription factors, with the latter having been identified in a wide range of plant species. NAC transcription factors possess a highly conserved NAC domain of approximately 150 amino acids at the beginning of the protein sequence, while their transcriptional regulatory region at the end of the sequence exhibits significant variability [6]. There are 117, 151, 269, 263, and 169 NAC genes in *Arabidopsis thaliana*, *Oryza sativa*, *Glycine max*, *Triticum aestivum*, and *Zea mays*, respectively [7]. Over the last decade, many NAC genes from various plant species, including crops, have served as suitable tools for the improvement of plant drought stress responses. ANAC019, ANAC055, and ANAC072/RD26 are important regulators in *Arabidopsis* drought stress response, as they confer drought tolerance to the plants by controlling the expression of stress-related genes [8]. In rice, transgenic plants overexpressing *SNAC1*, *OsNAC6*, and *ONAC022* were found to have increased the expression of stress-responsive genes and enhanced drought tolerance [9,10,11]. Using a genome-wide association study (GWAS), a 108 bp insertion was identified in the *TaNAC071-A* promoter that is associated with drought tolerance. This enhanced the expression of *TaNAC071-A* and generated drought tolerance in wheat [12]. In tomato, the silencing of the *SINAC4* gene reduced drought tolerance [13]. Transgenic plants overexpressing *GmNAC8* from soybean have been found to enhance drought resistance in *Arabidopsis* [14]. Overexpression of *SlNAC4* from *Suaeda liaotungensis* enhances the salt and drought tolerance of transgenic *Arabidopsis* [15]. The transcription factor RcNAC091 plays a positive role in the drought response of rose [16]. MdNAC29, on the other hand, has been found to reduce drought resistance in apple by directly repressing the expression of *MdDREB2A* [17]. Both *JUNGBRUNNEN1* and *SINAC6* play positive regulatory roles in the tomato drought response, increasing their expression and, thus, conferring enhanced drought tolerance to the plant [18,19]. The above studies collectively indicate that NAC transcription factors play a significant role in plant drought resistance processes. So far, there have been reports on the functions of certain NAC transcription factors in cotton. The NAC transcription factor GhFSN1 plays a positive regulatory role in secondary wall development in cotton fiber cells, while GhFSN5 functions as a negative regulator [20,21]. Suppressing the expression of *GhNAC82* effectively retards the aging process of cotton leaves, suggesting that this gene is involved in the regulation of leaf senescence in cotton [22]. Several NAC transcription factors have also been shown to be involved in cotton drought response. GhirNAC2 enhances the drought tolerance of cotton by regulating ABA biosynthesis [23]. Downregulating *GhJUB1L1* expression compromises drought tolerance in cotton [24]. Overexpression of cotton *GhNAC072* enhances drought and salt stress tolerance in transgenic *Arabidopsis* [25]. There are a total of 299 NAC genes in the cotton genome [26]. Nevertheless, the functions of most are still unclear, thereby necessitating further investigation.

Cotton (*Gossypium*) is a globally cultivated, significant cash crop, renowned for its fiber which serves as a vital raw material for the global textile industry. Additionally, cotton also acts as a source of oil, food, and various other industrial raw materials [27,28]. However, cotton often suffers from drought stress during its lifetime, which seriously affects its growth and development, thus reducing its quality and yield [29]. Research has found that 42% of cotton seed yield and 55% of cotton biological yield have been reduced due to drought stress [30,31].

Researching and identifying drought-responsive genes in cotton would establish a fundamental basis for the utilization of genetic engineering techniques to enhance drought resistance in cotton. In the current study, we identified a cotton NAC transcription factor, *GhNAC2-A06*, that was induced by PEG treatment, drought stress, and ABA treatment. We then functionally validated its role in cotton drought response using VIGS. We observed that inhibiting the expression of *GhNAC2-A06* increased drought sensitivity in cotton. Additionally, with the drought stress treatment, the transcription level of some drought-stress-responsive genes was reduced in *GhNAC2-A06* silenced cotton. Therefore, our results indicate that *GhNAC2-A06* acts as a regulator in the cotton drought stress response.

## 2. Results

### 2.1. Molecular Characterization of GhNAC2-A06

**Figure 1:**
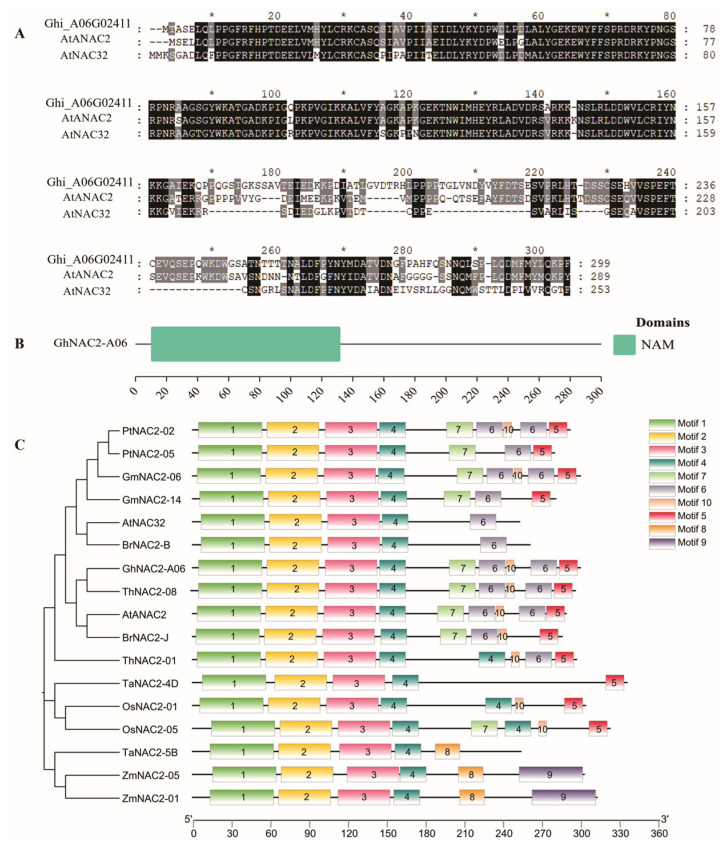
Molecular characterization of GhNAC2-A06. (**A**) Sequence alignment of GhNAC2-A06 with its *Arabidopsis thaliana* homologs. (**B**) Conserved domain analysis of GhNAC2-A06. (**C**) Phylogenetic analysis and conserved motifs of the GhNAC2-A06 protein and NAC proteins from other plants (*Arabidopsis thaliana*, *Populus trichocarpa*, *Glycine max*, *Brassica rapa*, *Theobroma cacao*, *Triticum aestivum*, *Oryza sativa*, and *Zea mays*).

We identified an NAC transcription factor *Ghi_A06G02411* from the cotton genome. Multiple sequence alignment analysis showed that it shared the highest homology with *AtANAC2* of the *Arabidopsis* genome (Figure 1A). Therefore, we named *Ghi_A06G02411* as *GhNAC2-A06* based on its chromosomal location and its *Arabidopsis* homolog. GhNAC2-A06 contained a typical N-terminal NAC domain (NAM), confirming that it is a NAC transcription factor (Figure 1B). The coding sequence (CDS) of *GhNAC2-A06* spanned 900 base pairs, encoding a protein comprising 300 amino acids. The protein of GhNAC2-A06 had a molecular weight of 33.88 KDa and a theoretical isoelectric point of 6.63 (Table S1). To understand the phylogenetic relationship of *GhNAC2-A06*, an unrooted phylogenetic tree was constructed using GhNAC2-A06 and AtANAC2 homologs in other species. Meanwhile, we analyzed their conservative motifs. As shown in Figure 1C, GhNAC2-A06 exhibited the highest similarity to cacao *ThNAC2-08*, with an identical motif composition. GhNAC2-A06 also displayed high similarity with and identical motif composition to AtANAC2 and BrNAC2-J. The N-terminus of all these NAC proteins contains motifs 1, 2, 3, and 4, indicating that they are part of the conserved domain NAM in NAC proteins (Figure 1C). Additionally, we observed that the NAC genes of monocotyledons and dicotyledons were grouped into separate clusters, indicating the independent evolution of NAC genes after the divergence of monocotyledons and dicotyledons (Figure 1C). According to the *cis*-element analysis, the promoter of *GhNAC2-A06* was found to contain multiple *cis* elements associated with an abiotic stress response (Figure S1). This suggests that GhNAC2-A06 may play a role in the response to abiotic stress in cotton.

### 2.2. Expression Pattern of GhNAC2-A06 under Drought Stress and ABA Treatment

**Figure 2:**
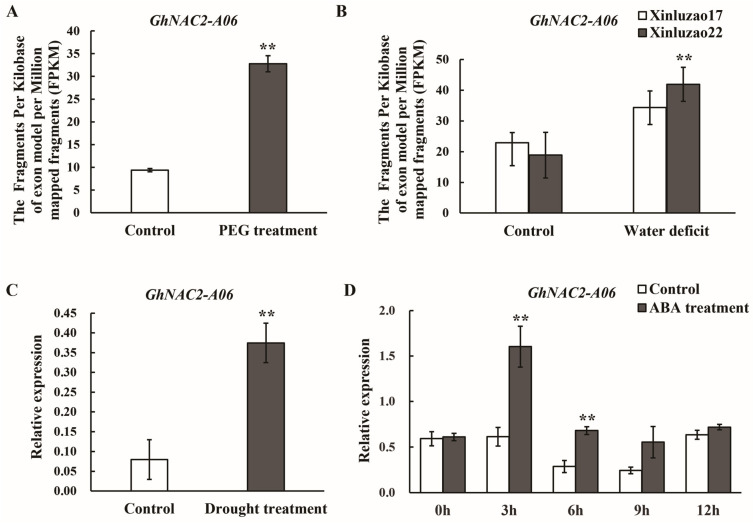
Expression pattern of *GhNAC-A06*. (**A**) Expression pattern of *GhNAC2-A06* under the PEG treatment. The PEG-treatment-related transcriptome data were obtained from our lab. (**B**) The expression of *GhNAC2-A06* in drought sensitive (Xinluzao 17) and drought tolerant (Xinluzao 22) varieties under water deficit conditions. The transcriptome data were download from NCBI SRA database. (**C**) Expression pattern of *GhNAC2-A06* investigated using qPCR under drought conditions. (**D**) The qPCR analysis of the transcription levels of *GhNAC2-A06* under ABA treatment. The data presented represent the mean values and standard errors (indicated by error bars) derived from three independent experiments. The statistical analysis was performed using the student’s *t*-test, with asterisks (*) denoting significant differences: ** *p* < 0.01.

Transcriptome data from our lab showed that the expression of *GhNAC2-A06* increased under the PEG treatment (Figure 2A). In addition, further transcriptome data indicated that, under water deficit conditions, the transcription of *GhNAC2-A06* was upregulated in both the drought-tolerant variety Xinluzao 22 and the drought-sensitive variety Xinluzao 17, and the upregulation degree of *GhNAC2-A06* was higher in the drought-tolerant variety than in the drought-sensitive variety (Figure 2B). These results suggest a potential role of *GhNAC2-A06* in drought stress response. To further verify this deduction, quantitative PCR (qPCR) was carried out to detect the expression of *GhNAC2-A06* in the leaves of seedlings under normal growth conditions and under the drought treatment. The qPCR results showed an increase in *GhNAC2-A06* expression under the drought treatment (Figure 2C). Therefore, the above results suggest that *GhNAC2-A06* is relevant to the drought response of cotton.

The promoter of the *GhNAC2-A06* gene contains multiple ABRE *cis*-acting elements (Figure S1). To investigate whether the *GhNAC2-A06* gene could respond to the ABA signal, the expression pattern of *GhNAC2-A06* under the ABA hormone treatment was examined using a qPCR technique. The results showed that *GhNAC2-A06* was significantly upregulated at 3 h and 6 h after exogenous ABA treatment, and there was no significant change after 12 h (Figure 2D). The results demonstrate that the *GhNAC2-A06* gene was induced by the ABA hormone treatment, suggesting that it is involved in the ABA hormone signaling pathway.

### 2.3. Silencing of GhNAC2-A06 Compromises the Tolerance of Cotton to Drought Stress

**Figure 3:**
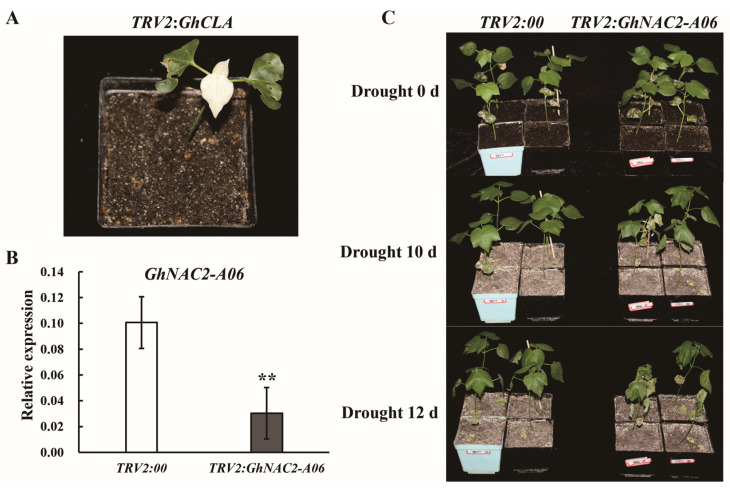
Phenotypic evaluation of cotton plants with the *GhNAC2-A06* gene silenced under drought stress. (**A**) Albino characteristics observed in the positive control cotton plants. (**B**) qPCR examination of the expression of *GhNAC2-A06* in *TRV2:00* and *TRV2:GhNAC2-A06* plants. The mean values and error bars denoting the standard deviation were calculated from three independent experiments. Asterisks represent the student’s *t*-test in statistical analysis for significant differences: ** *p* < 0.01. (**C**) Phenotypic analysis of the *TRV2:00* and *TRV2:GhNAC2-A06* plants after withholding water for 10 days and 12 days.

In order to investigate the involvement of *GhNAC2-A06* in the cotton plant response to drought stress, we carried out a VIGS. Plants infected with *TRV2:GhCLA* exhibited the albino phenotype, suggesting that the expression of the target gene had been suppressed (Figure 3A). Subsequently, we investigated the *GhNAC2-A06* expression levels in the control (*TRV2:00*) and the *GhNAC2-A06* silenced plants (*TRV2:GhNAC2-A06*) through qPCR. Compared to the control, the expression of *GhNAC2-A06* was significantly downregulated in the *TRV2:GhNAC2-A06* plants (Figure 3B). In addition, we also investigated the expression levels of genes with high similarity to *GhNAC2-A06*, including *Ghi_D02G03756* and *Ghi_A02G03216*. The results show that the expression of *Ghi_D02G03756* and *Ghi_A02G03216* did not differ between the *TRV2:GhNAC2-A06* plants and the control plants (Figure S3B,C). Afterward, the seedlings that were four weeks old were exposed to the drought treatment. Before the drought treatment, we observed no significant differences in the phenotype between control and silenced plants. After withholding water for 12 days, the *TRV2:00* plants showed better growth status than the *TRV2:GhNAC2-A06* plants. Furthermore, we noticed that the silenced plants exhibited a higher degree of wilting leaves compared to the control (Figure 3C).

Reducing stomatal opening is a crucial mechanism that plants employ to minimize water loss when experiencing drought stress [32]. Therefore, the stomatal apertures of control plants and silenced plants were observed under normal conditions and under the drought treatment. Under normal conditions, the stomatal aperture of the *GhNAC2-A06* silenced plants was similar to that of the control plants. However, under drought stress, the stoma of *GhNAC2-A06* silenced plants exhibited larger openings compared to those of the controls (Figure S2A,B). These results indicate that silencing *GhNAC2-A06* compromises cotton tolerance to drought stress.

### 2.4. Effect of Silencing GhNAC2-A06 on the Physiological and Biochemical Characteristics of Cotton

**Figure 4:**
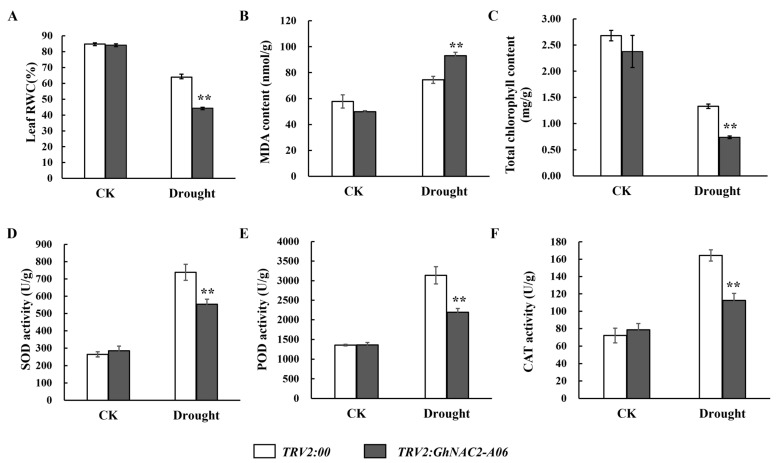
Analysis of the physiological indexes in plants with silenced *GhNAC2-A06* and in control plants under normal watering conditions (CK) and drought treatment conditions. (**A**) The leaf RWC (relative water content). (**B**) MDA content. (**C**) Chlorophyll content. (**D**) SOD activity. (**E**) POD activity. (**F**) CAT activity. The mean values and error bars indicating the standard deviation were computed based on three separate experiments. Asterisks were used to represent the student’s *t*-test in statistical analysis to indicate significant differences: ** *p* < 0.01.

By regulating their own physiological and biochemical characteristics, plants have the ability to improve their tolerance to drought stress [33]. In order to examine the potential impact of silencing the *GhNAC2-A06* gene on the physiological and biochemical traits of cotton, we conducted measurements of various physiological and biochemical parameters in both the control group and the group with *GhNAC2-A06* silenced plants. No significant variation in leaf relative water, MDA, and chlorophyll content was observed between the *TRV2:00* and *TRV2:GhNAC2-A06* plants under normal growth conditions (Figure 4A–C). Moreover, the activities of SOD, POD, and CAT enzymes were found to be similar in both *TRV2:00* and *TRV2:GhNAC2-A06* plants, with no significant differences (Figure 4D–F). However, under drought stress, *TRV2:00* plants showed a higher leaf relative water and chlorophyll content and a lower MDA content than the *TRV2:GhNAC2-A06* plants (Figure 4A–C). This indicates that the control plants exhibited lower cellular damage in comparison to the plants with *GhNAC2-A06* silencing. Under drought stress conditions, the *TRV2:00* plants showed significantly increased levels of SOD, POD, and CAT enzyme activity compared to the *TRV2:GhNAC2-A06* plants (Figure 4D–F). This suggests that the silencing of the *GhNAC2-A06* compromised the antioxidant capacity of cotton. As a result, it was found that the growth condition of the control plants was superior to that of the target gene-silenced plants under drought stress (Figure 3).

### 2.5. GhNAC2-A06 Affects the Expression of Some Drought-Stress-Related Genes in Cotton

**Figure 5:**
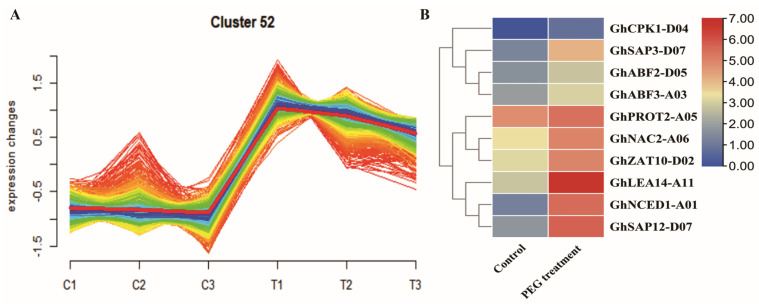
Expression trend clustering analysis. (**A**) The expression trend of the module to which *GhNAC2-A06* belongs under the PEG treatment. C1, C2, and C3 correspond to the three replicates of the control group, while T1, T2, and T3 correspond to those of the PEG treatment group. (**B**) Expression patterns of the known drought-responsive genes in the module having *GhNAC2-A06* under the PEG treatment.

Transcription factors play a crucial role in plant response to drought by controlling the expression of genes associated with drought stress [34]. To screen for drought-responsive genes that may be regulated by *GhNAC2-A06*, we conducted expression module clustering analysis on the transcriptome data. We found that *GhNAC2-A06* belonged to the expression module 52, which showed upregulated gene expression under the PEG treatment (Figure 5A). Subsequently, we selected several previously reported drought stress-responsive genes from module 52 for further analysis (Figure 5B and Table S2).

**Figure 6:**
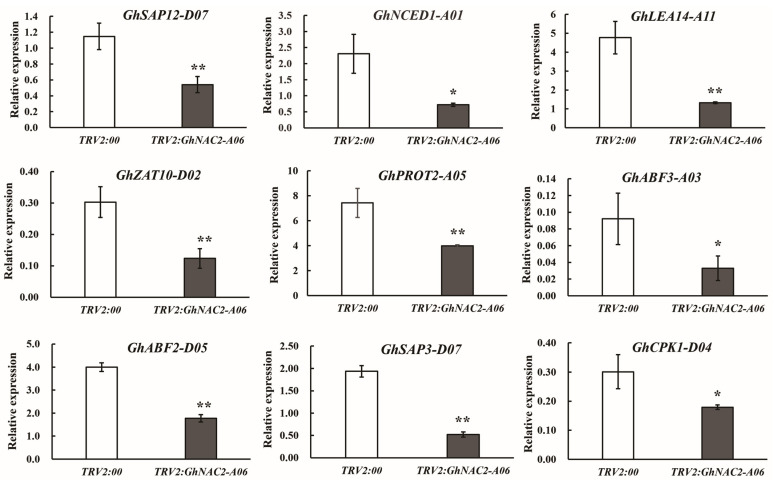
Expression analysis of the drought-stress-related genes. The qPCR analysis was conducted to measure the expression levels of drought-stress-related genes in both *TRV2:00* control and *GhNAC2-A06*-silenced plants. *GhHIS3* was used as the internal control. The mean values and standard errors were calculated from three independent experiments. Asterisks indicate the statistical significance assessed by the student’s *t*-test: * *p* < 0.05, ** *p* < 0.01.

We analyzed the expression of these genes that responded to drought stress in both the control plants and the plants with the silenced *GhNAC2-A06* gene. As shown in Figure 6, the gene expression levels of *GhSAP12-D07*, *GhNCED1-A01*, *GhLEA14-A11*, *GhZAT10-D02*, *GhPROT2-A05*, *GhABF3-A03*, *GhABF2-D05*, *GhSAP3-D07*, and *GhCPK1-D04* were reduced in the *TRV2:GhNAC2-A06* plants compared to the *TRV2:00* plants when exposed to drought stress. Hence, these findings indicate that *GhNAC2-A06* plays a role in the cotton plant response to drought by regulating the expression of genes involved in drought response.

## 3. Discussion

NAC transcription factors are specific to plants and play a critical role in the response to abiotic stress [35,36]. In rice, overexpression of NAC transcription factors (*OMTN2*, *OMTN3*, *OMTN4*, and *OMTN6*) reduced drought tolerance in plants [37]. Mao et al. identified a maize drought-stress-responsive gene, *ZmNAC111*, through a GWAS. Enhancing the expression of *ZmNAC111* improved maize drought tolerance [38]. In soybean, *GmNAC12*-overexpressing plants exhibited stronger drought tolerance compared to the wild type [39]. *TaNAC5D-2* has a positive effect on the drought tolerance of plants and expressing it in *Arabidopsis* reduced water loss under drought stress [40]. However, TaSNAC4-3D has a negative impact on wheat drought stress response [41]. In cowpea, overexpressing *NAC1* and *NAC2* genes provided transgenic plants with enhanced drought tolerance [42].

In the current study, we identified and isolated a NAC transcription factor, named *GhNAC2-A06*, from the cotton genome. The N-terminus of GhNAC2-A06 possesses a NAM domain. Multiple sequence alignment and evolutionary analysis showed that *GhNAC2-A06* has the highest genetic relationship with *ThNAC2-08* and *AtANAC2*, indicating that *GhNAC2-A06* belongs to the ATAF subfamily of NAC transcription factors (Figure 1). The transcription factors of the ATAF subfamily are crucial in enhancing tolerance to drought stress. For example, the overexpression of the *CaNAC46* gene in the ATAF subfamily has been found to enhance drought tolerance in genetically modified *Arabidopsis* [43]. In addition, we also identified multiple ABA and drought-responsive *cis* elements in the promoter region of *GhNAC2-A06* (Figure S1). Therefore, we posited that *GhNAC2-A06* might have a function in addressing drought stress.

To further ascertain whether *GhNAC2-A06* is associated with drought response in cotton, we investigated the expression pattern of *GhNAC2-A06*. We found that drought stress (18% PEG-6000, or water withholding) and ABA treatment significantly induced the expression of *GhNAC2-A06*, suggesting its involvement in the drought response process of cotton (Figure 2A–D). ABA is the core regulatory factor that regulates plant drought response. It optimizes plant water utilization strategies and enhances plant tolerance to drought stress by controlling the expression of a series of stress-related genes [44]. The expression of *RcNAC091* is induced by ABA, and it plays a positive regulatory role in the rose drought response process [16]. ABA induces the transcription of *VvNAC08*, and overexpression of *VvNAC08* enhances drought tolerance in *Arabidopsis* [45]. In the current study, the transcripts of *GhNAC2-A06* significantly accumulate under ABA treatment, further suggesting that *GhNAC2-A06* may play a role in the drought response of cotton.

Cotton is an allotetraploid plant formed by the fusion of two diploid subgenomes, AA and DD [46]. Most genes in the A subgenome have orthologous genes in the D subgenome, and the sequences of these homologous genes between the A and D subgenomes are highly similar [46]. It is difficult to distinguish and selectively silence orthologous genes between the A and D subgenomes using VIGS technology [47]. During our research on the functionality of *GhNAC-A06* using VIGS, we found that the expression of *Ghi_D06G02306* was also downregulated in *TRV2:GhNAC2-A06* plants. This gene is a homologous gene of *GhNAC2-A06* in the D subgenome. At the same time, we found that the expression level of the *Ghi_D06G02306* gene was extremely low in the leaves of cotton plants (Figure S3D). Therefore, the reduced drought tolerance observed in the *TRV2:GhNAC2-A06* plants is caused by the silencing of the *GhNAC2-A06* gene, rather than *Ghi_D06G02306*.

We observed that the degree of leaf wilting in plants with silenced *GhNAC2-A06* was more severe compared to the control plants after the drought treatment, indicating that the silencing of *GhNAC2-A06* reduces the ability of cotton to withstand drought stress (Figure 3B). This finding not only proves the significant role of *GhNAC2-A06* in the drought stress response of cotton, but also provides new support for the critical involvement of NAC transcription factors in plant drought stress response. Under conditions of drought stress, plants frequently decrease the extent of stomatal opening as a means of restricting water loss within the cells [32]. Overexpression of *ZmNAC48* promotes stomatal closure in *Arabidopsis* under drought stress [48]. By facilitating the closure of stomata, ZmNAC20 enhances the ability of plants to withstand drought stress [49]. In this study, our data show that the stomata of the *GhNAC2-A06* silenced cotton plants opened wider than those of the controls under drought stress, suggesting that silencing *GhNAC2-A06* influences the closure of stomata (Figure S2). Both our results and previous studies have demonstrated that NAC transcription factors can influence plant drought stress tolerance by regulating the stomatal aperture.

The occurrence of abiotic stress often leads to an overproduction of reactive oxygen species (ROS), resulting in severe damage to plant cells [50]. Plants can remove excessive ROS through the ROS scavenging system to maintain a low ROS content under abiotic stress [50]. Previous studies have shown that NAC can influence the accumulation levels of ROS in plants by modulating the antioxidant defense system [51]. Overexpressing *OsNAC3* in rice increases the expression level of ROS-scavenging genes, thereby decreasing ROS accumulation in plants under drought stress [52]. AtNAC075 was found to affect ROS accumulation in plants by regulating the expression of ROS-scavenging genes, including genes related to CAT, ascorbate peroxidase (APX), and SOD [53]. Overexpressing *ThNAC7* showed increased ROS-scavenging capabilities, which involved enhanced SOD and POD activities in transgenic plants [54]. Silencing *GhNAC2-A06* resulted in higher accumulation of MDA and reduced CAT, SOD, and POD activity in cotton, thereby suggesting a reduced ROS-scavenging ability of the *GhNAC2-A06* gene-silenced plants, ultimately meaning that plants are unable to carry out normal metabolism and growth (Figure 4B–F). These findings align with prior research demonstrating the involvement of NAC genes in enhancing drought stress tolerance through the regulation of ROS-scavenging systems. Chlorophyll content is also an important indicator for the evaluation of plant drought resistance [55]. Under drought conditions, the chlorophyll content of *GhNAC2-A06*-silenced plants is significantly lower compared to that of the control plants, further indicating that the silencing of *GhNAC2-A06* weakens the drought tolerance of cotton.

Plant transcription factors are integral in mediating diverse stress responses through their control over the expression of target genes associated with stress [5]. Previous research has provided evidence that NAC transcription factors perform vital functions in enhancing drought tolerance. They achieve this by directly or indirectly impacting the expression of genes associated with stress responses [9,16,18]. Transcriptomic analysis reveals that multiple drought response genes were upregulated in transgenic lines overexpressing *ZmNAC20* [49]. The expression of certain drought-responsive genes was upregulated in transgenic plants overexpressing *VvNAC17* [56]. AtJUB1 increases drought tolerance in tomatoes, though directly stimulating the expression of *SlDREB1*, *SlDREB2*, and *SlDELLA* [18]. ONAC066 positively regulates rice response to drought by directly regulating the *OsDREB2A* expression [57]. Both OsNAC9 and ZmNAC55 act as positive regulators of the drought stress response, as they can promote transcription of the ABA-synthesis-related NCED genes [58,59]. Our data demonstrate that silencing *GhNAC2-A06* downregulates the expression levels of drought-responsive genes including *GhSAP12-D07*, *GhNCED1-A01*, *GhLEA14-A11*, *GhZAT10-D02*, *GhPROT2-A05*, *GhABF3-A03*, *GhABF2-D05*, *GhSAP3-D07*, and *GhCPK1-D04*. This further indicates that NAC transcription factors regulate plant drought response by modulating the expression of drought-responsive genes.

## 4. Materials and Methods

### 4.1. Plant Material and Treatment

The cotton variety Xinluzao 42 was obtained from the Cotton Germplasm Resources Research Office of Xinjiang Academy of Agricultural Sciences. Cotton seeds were placed on moistened filter paper for germination. The germinated seeds were then planted in a bowl containing nutrient soil vermiculite (1:3; *v*/*v*). The cultivation of the specimens took place in a light chamber maintained at a constant temperature of 25–28 °C, under a photoperiod of 16 h light and 8 h darkness, with a relative humidity of 70%. To simulate drought stress treatment, 18% PEG-6000 was used and the leaves of cotton seedlings were collected after 4 h of PEG-6000 treatment. The drought treatment was performed on cotton seedlings after they had grown a third true leaf. Cotton was subjected to moderate drought stress; when the relative soil moisture content in the drought treatment group dropped below 50%, cotton leaves were collected. We weighed a certain mass of ABA powder, added a trace volume of anhydrous ethanol to fully dissolve the powder, and then diluted it with water to a final concentration of 100 μM ABA hormone culture solution. Cotton plants with both true leaves fully expanded and in the same growth condition were selected, and the prepared ABA hormone culture solution was uniformly sprayed on the cotton leaves, while the control group was treated with an equal volume of anhydrous ethanol; leave samples were taken 0 h, 3 h, 6 h, 9 h, and 12 h after ABA treatment.

### 4.2. Bioinformatics Analysis of GhNAC2-A06

The protein sequences of *GhNAC2-A06* and *AtNAC2* were downloaded from Cotton MD (http://yanglab.hzau.edu.cn/CottonMD/, accessed on 18 April 2023) and TAIR (https://www.arabidopsis.org/database, accessed on 19 April 2023), respectively. The MUSCLE5 software was used for multiple sequence alignment. The NAC protein sequences from other plants were obtained from the phytozome database (https://phytozome-next.jgi.doe.gov/, accessed on 21 April 2023). Next, we conducted phylogenetic analysis using MEGA7.0 software with the Neighbor-Joining method, which had 1000 bootstrap values. The protein sequences of *GhNAC2-A06* and NAC proteins from other species were submitted to the MEME database (https://meme-suite.org/meme/tools/meme, accessed on 25 April 2023) for analysis of the composition of conserved motifs [60]. The results from the phylogenetic analysis and the motif analysis were submitted to TBtools2.003 software to have both displayed together. The 2000 bp sequences upstream of *GhNAC2-A06* translation start codon were extracted from the Cotton MD database as the promoter of *GhNAC2-A06.* Then, the promoter of *GhNAC2-A06* was submitted to the PlantCARE database (http://bioinformatics.psb.ugent.be/webtools/plantcare/html/, accessed on 26 April 2023) to analyze the *cis* elements distribution.

### 4.3. Expression Analysis

The fragments per kilobase per million reads (FPKM) value of *GhNAC2-A06* was extracted from the transcriptome data under the PEG-treated condition. The transcriptome data under drought stress were downloaded from the NCBI SRA database (https://www.ncbi.nlm.nih.gov/sra/?term=PRJNA776142, accessed on 28 April 2023) [61]. The reads of the transcriptome data were aligned to the *Gossypium hirsutum* reference genome (v2.1) using Hisat2, and gene expression quantification was performed with HTSeq [62]. Cotton plants at the three true leaf stage underwent a water-withholding treatment. When the soil relative water content fell below 50%, cotton leaves were collected for RNA extraction. The extraction of total RNA from the leaves was carried out using the Plant Total RNA Isolation Kit Plus (Foregene Co., Ltd., Chengdu, China), following the manufacturer’s instructions. The extracted RNA (approximately 2 μg) was reverse transcribed into first-strand cDNA using the RT EasyTM II (With gDNase Kit) (Foregene Co., Ltd., Chengdu, China). A list of all the gene-specific primers used in the qPCR experiment is provided in Table S2. The 2^−∆∆Ct^ method was used to calculate the relative expression value of candidate genes. Cotton *GhHis3* was used as an endogenous standard control. All experiments were conducted with three biological replicates.

### 4.4. Vector Construction and Procedure for VIGS in Cotton

The VIGS experiment followed the methodology previously described by other researchers [63]. To construct the *TRV2:GhNAC2-A06* recombinant plasmids, 400 bp fragments of *GhNAC2-A06* were amplified from the cDNA library using PCR and then inserted into the TRV2 vector. The primer for constructing the vector is listed in Table S2. The *TRV1*, *TRV2:00*, *TRV2:GhNAC2-A06*, and *TRV2:GhCLA* plasmids were introduced into the *Agrobacterium tumefaciens* GV3101 strain. Mixed strains of *Agrobacterium tumefaciens* containing *TRV1*, *TRV2:00*, and *TRV2:GhNAC2-A06* or *TRV2:GhCLA* in equal proportions were incubated at 28 °C for 3 h. To generate the control (*TRV2:00*) and *GhNAC2-A06* silenced (*TRV2:GhNAC2-A06*) cotton plants, the agrobacterium mixture was injected into the cotyledons of 10-day-old cotton seedlings using a syringe. *TRV2:GhCLA* served as the positive control, where the albino phenotype indicated successful gene suppression. Subsequently, the expression of *GhNAC2-A06* was detected in the *TRV2:GhNAC2-A06* plants and in the *TRV2:00* controls using qPCR. Lastly, the cotton plants with successfully suppressed target genes and control plants were subjected to a drought treatment.

### 4.5. Survey on Physiological Parameters of Cotton Plants

The quantitative determination of the MDA content, chlorophyll content, proline content, POD enzyme activity, SOD enzyme activity, and CAT enzyme activity in leaves was performed according to the instructions provided by the manufacturer of the MDA detection kit, chlorophyll detection kit, proline detection kit, POD assay kit, SOD assay kit, and CAT assay kit (Nanjing Jiancheng Bioengineering Institute, Nanjing, China), respectively. The second true leaves of the *GhNAC2-A06*-silenced plants and of the control plants were selected before and after 12 days of drought treatment. The leaf RWC was determined using the drying method, with three sample replicates for each treatment. After 12 days of drought treatment for both the control group and the silenced group, the epidermis of cotton leaves was peeled off, and the stomatal aperture was observed using an inverted fluorescence microscope of Leica DMi8 (Leica Microsystems Limited Company, Vitzla, Germany). This experiment was repeated three times, with more than 30 stomata detected each time. The ratio of stomata length to width was measured using CorelDRAW2019 software.

### 4.6. Expression Trend Clustering Analysis

A total of six sets of transcriptome data were used to perform the expression trend cluster analysis under PEG-treated conditions. The log (FPKM+1) was utilized as input data for the R package Mfuzz in order to conduct trend clustering analysis. Afterwards, we functionally annotated the genes of the cluster module containing *GhNAC2-A06*, aiming to identify the known drought responsive genes. The expression of these drought response genes was evaluated in the control samples and in the cotton plants with silenced target genes using qPCR analysis.

## 5. Conclusions

In this study, we cloned and characterized a cotton NAC transcription factor, *GhNAC2-A06.* The expression of *GhNAC2-A06* was induced by drought stress (18% PEG-6000 treatment, or water withholding) and ABA treatment. The growth status of *GhNAC2-A06* gene-silenced plants was poorer than that of the control plants under drought stress, and the wilting severity of *GhNAC2-A06* gene-silenced plant leaves was more severe. After the drought treatment, the MDA content of *GhNAC2-A06*-silenced plants was higher than that of the controls, while the leaf RWC, chlorophyll content, and activities of SOD, POD, and CAT enzymes were lower than those observed in the controls. Furthermore, the expression level of *GhABF2-D05*, *GhCPK1-D04*, *GhLEA14-A11*, *GhNCED1-A01*, *GhPROT2-A05*, *GhSAP3-D07*, *GhSAP12-D07*, and *GhZAT10-D02* was downregulated in *GhNAC2-A06* silenced plants compared to the controls under drought stress. The data above indicate that the silencing of *GhNAC2-06* increased the sensitivity of cotton to drought stress. This study provides valuable information and deepens our understanding of the function and regulatory mechanisms of NAC transcription factors.

## Data Availability

Transcriptome data under the PEG treatment are available from the corresponding author (18910445207@163.com) upon reasonable request.

## Supplementary Materials

The following supporting information can be downloaded at: https://www.mdpi.com/article/10.3390/plants12213755/s1, Figure S1. Analysis of *cis*-elements in the *GhNAC2-A06* gene promoter; Figure S2. The stomatal aperture of *TRV2:00* and *TRV2: GhNAC-A06* plants after the drought treatment; Figure S3. qPCR analysis of *Ghi_D06G02306*, *Ghi_D02G03756*, *Ghi_A02G03216*, and *GhNAC2-A06*; Table S1. Basic information of *GhNAC2-A06*; Table S2. Primers used in this study; Table S3. Gene IDs used in this study.
