# Supplementary material for: The Functions of an NAC Transcription Factor, GhNAC2-A06, in Cotton Response to Drought Stress
Source: Plants (Basel). 2023 Nov 2;12(21):3755. doi: 10.3390/plants12213755 (PMC10649604; doi:10.3390/plants12213755)
Supplement: Supplementary file 1 [file plants-12-03755-s001.zip › Supplementary figure.pdf]

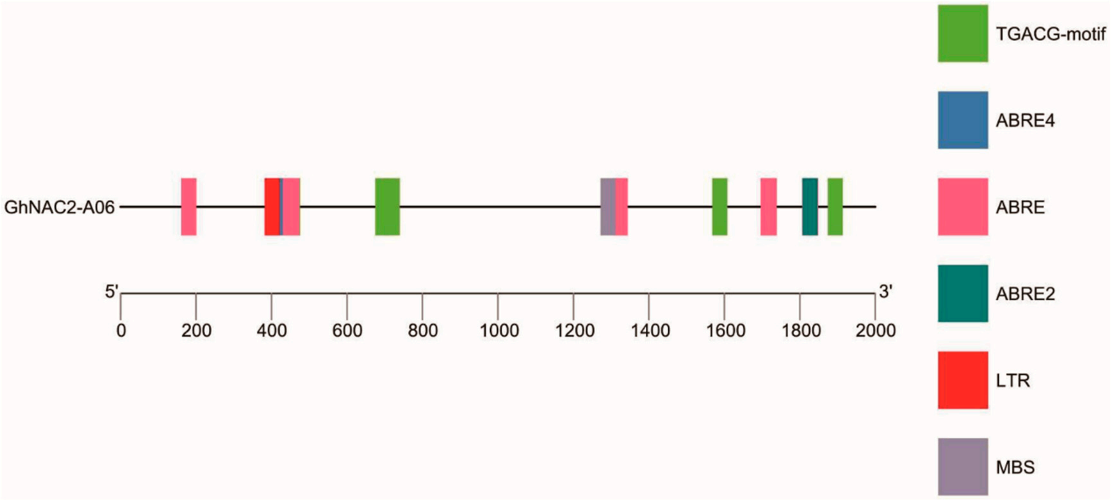

Figure S1. Analysis of *cis*-elements in *GhNAC2-A06* gene promoter. Diverse colors were used for representing different *cis*-elements as shown on the right side.

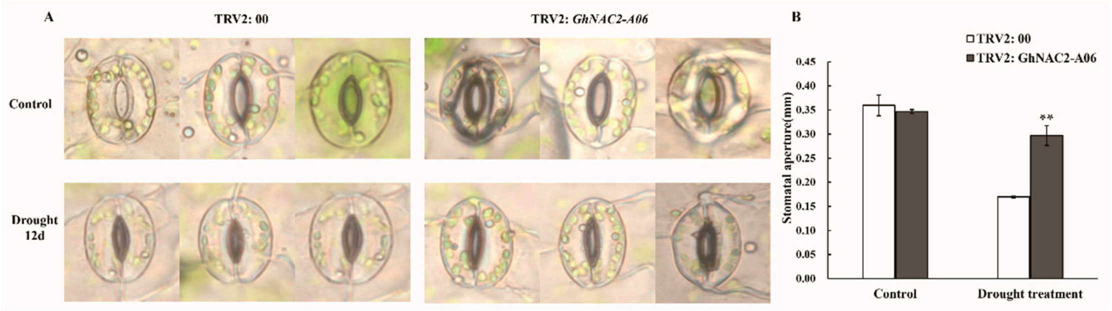

Figure S2. The stomatal aperture of *TRV2: 00* and *TRV2: GhNAC-A06* plants after drought treatment. (A) Stomatal images of control and silent plants under normal growth conditions (control) and drought treatment. (B) Comparative stomatal aperture measurements (ratio of width to length). Asterisks represent the Student's *t*-test in statistical analysis for significant differences: $**p < 0.01$.

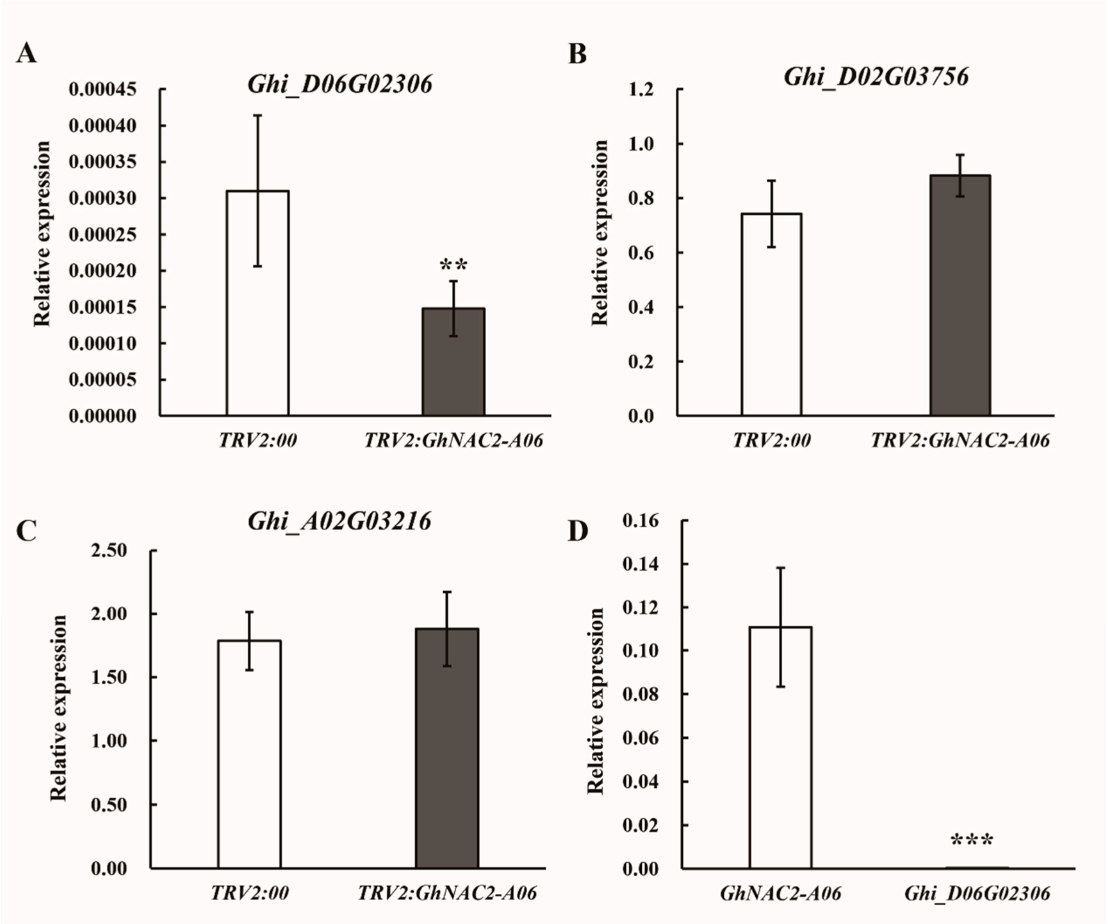

Figure S3. QPCR analysis of *Ghi_D06G02306*, *Ghi_D02G03756*, *Ghi_A02G03216* and *GhNAC2-A06*. (A-C) Transcription levels of *Ghi_D06G02306 Ghi_D02G03756* and *Ghi_A02G03216* in the *TRV2:00* and *TRV2:GhNAC2-A06* plants. (D) Transcription levels of *GhNAC2-A06* and *Ghi_D06G02306* in the leaves of *TRV2:00* plants. The mean values and standard errors were calculated from three independent experiments. Asterisks indicate the statistical significance assessed by the Student's *t*-test: $^{**}p < 0.01$, $^{***}p<0.001$.
