# Supplementary material for: The Functions of an NAC Transcription Factor, GhNAC2-A06, in Cotton Response to Drought Stress
Source: Plants (Basel). 2023 Nov 2;12(21):3755. doi: 10.3390/plants12213755 (PMC10649604; doi:10.3390/plants12213755)
Supplement: Supplementary file 1 [file plants-12-03755-s001.zip › Supplementary table.pdf]

Table S1. Basic information of GhNAC2-A06.

Gene name	Gene symbol	Lengh(aa)	MW(kDa)	pI	Chr	Chr location
GhNAC2-A06	Ghi_A06G02411	300	33.88	6.63	A06	7,938,618-7,940,149

Table S2. Primers used in this study.

Gene	Primer
GhNAC2-A06-F	CCCGAATTCTTGCCGGATTTATAACAAGAAAG
GhNAC2-A06-R	CTTCTCGAGCAGCTGATTATTACTCTGGAAAT
GhNAC2-A06-RT-F	TGTTTTACGCTGGGAAGGCA
GhNAC2-A06-RT-R	CCGCCTCCGGTTTATCCCTA
GhABF2-D05-RT-F	ACGGTATTGGAGGTGGAGGA
GhABF2-D05-RT-R	CCCACCAAAGAACCCCTCAT
GhABF3-A03-RT-F	TCGGCTTTAGCTCTTGGGTT
GhABF3-A03-RT-R	GGACTAGCGAGCTGTGTTGC
GhCPK1-D04-RT-F	ATCTCATGGCAGCCTTCTCG
GhCPK1-D04-RT-R	AGCCTAGGATTGCAAGATCAG
GhLEA14-A11-RT-F	GCGCTAAGGTCTCTGTCTCC
GhLEA14-A11-RT-R	TCAATGTCCCAATCTGCACCA
GhNCED1-A01-RT-F	TCACCCCAAAGTTGATCCACAA
GhNCED1-A01-RT-R	TTTCAGTGATGGCGAAGTCGT
GhPROT2-A05-RT-F	GCCTTGCAGCTTAACAGCC
GhPROT2-A05-RT-R	AGACATTGAGCCAATGCCAGA
GhSAP12-D07-RT-F	CCAACTTGCCCCGTTAAAAGA
GhSAP12-D07-RT-F	CTCGTTCCAGCTTCCTCCAG
GhSAP3-D07-RT-F	AGATGTAAAGCACCGCCACA
GhSAP3-D07-RT-R	GAAAAGGAAGGGAGGAGGGC
GhZAT10-D02-RT-F	ACTGCCCCTTACAGCAACAA
GhZAT10-D02-RT-R	CTTCTTCATTCGTCGGCGGT
Ghi_D06G02306-RT-F	CACCGCAAGGAAGCATTGG
Ghi_D06G02306-RT-R	CACCGCAAGGAAGCATTGG
Ghi_D02G03756-RT-F	AGAACAGCCTAAGGTTGGACG
Ghi_D02G03756-RT-R	TACGTAATCGTTACCCACCG
Ghi_A02G03216-RT-F	GTGCTGTGCCGGATTTACAA
Ghi_A02G03216-RT-R	GTTACCAATCGCCGTAGCTG
GhHIS3-F	TCAAGACTGATTTGCGTTTCCA
GhHIS3-R	GCGCAAAGGTTGGTGTCTTC

Table S3. Gene IDs used in this study.

Gene_ID	Gene_Name
Ghi_D05G07576	*GhABF2-D05*
Ghi_D13G01991	*GhABF3-A03*
Ghi_D04G05266	*GhCPK1-D04*
Ghi_A11G05011	*GhLEA14-A11*
Ghi_D01G01581	*GhNCED1-A01*
Ghi_A05G17771	*GhPROT2-A05*
Ghi_D07G12396	*GhSAP12-D07*
Ghi_D07G05926	*GhSAP3-D07*
Ghi_D02G11446	*GhZAT10-D02*
